# Revisiting ‘who gets in’: borders and migration management in the era of automation and AI in Canada

**DOI:** 10.1007/s00146-025-02602-5

**Published:** 2025-09-26

**Authors:** Danièle Bélanger, Gabriel Bergevin-Estable

**Affiliations:** Université Laval, Québec, Canada

**Keywords:** Migration management, Automation, AI, Canada, Discretion, Chinook

## Abstract

This article examines the transformation of borders with the integration of Automated Decision Support Systems, including Artificial Intelligence, in Canada's migration management since 2015. This study pursues the dual objectives of analyzing the introduction of ADSS and its impact on decision-making regarding immigration applications. The research focuses on the Chinook system, introduced in 2018 for temporary immigration files, and explores how these technologies influence the discretion of visa officers, who are the ultimate decision-makers. Through an in-depth content analysis of governmental documents, many obtained via Access to Information requests, this study provides unique insights into the use of automation and artificial intelligence in immigration application processing and decision-making. Through a fine grain analysis, this article argues that Automated Decision Support Systems significantly alter the process of determining 'who gets in' by transforming the exercise of human discretion, therefore reconfiguring the confines of borders. The conclusion discusses how this new era of Canada’s migration management has profound human and legal consequences that call for the need to balance technological advancements with the preservation of substantive decision-making processes to protect procedural fairness and access to justice for immigration candidates who appeal a refusal.

## Introduction

Previous research has examined the steady incorporation of AI technologies into migration management (McAuliffe et al. 2021). Authors warned against the pitfalls of these technologies to manage human migration including infringement of privacy, threats to human rights, reinforcement of unlawful practices, all of these fuelled by a vibrant high-tech migration control industry (Kinchin 2024; Molnar 2024; Molnar and Gill 2018). The study of AI systems in migration covers mostly border surveillance, risk assessment, and biometrics (Nalbandian and Dreher 2022). In parallel to these trends, public administrations managing national border entries and travel authorizations have developed automated decision support systems (ADSS) that assist bureaucrats to determine ‘who gets in’, developments which have received less attention than other technologies to date. Canada paved the way for the introduction of automation in assessing immigration with legislative changes introduced in 2015. Since then, an array of tools that involve partial or total automated decision-making systems has been introduced by Immigration, Refugees and Citizenship Canada (IRCC).

The Government of Canada claims that the introduction of these new software tools does not in any way change the way decisions are made (IRCC 2022c). Given this context, and harnessing the concept of administrative border, we ask: how do automation and AI systems affect the way discretionary decisions are made in Canadian immigration as to ‘who gets in’? This article first analyzes the introduction of Automated Decision Support Systems (ADSS), some of which utilize artificial intelligence, for processing immigration applications in Canada since 2015. Second, it examines how ADSS impact the decision-making process regarding 'who gets in.' By focusing on a specific tool, Chinook, introduced in 2018 to handle temporary immigration files, we document this transformation, by concentrating on the use of discretion among Canadian visa officers in this new technological workflow.

Discretion in immigration decision-making is embedded in Canada’s Immigration and Refugee Protection Act (IRPA), which stipulates an officer must be satisfied of conformity when approving a file (IRPA 2001, 11(1)). For temporary resident applications in particular, eligibility decisions are highly discretionary (Satzewich 2015). Previous research had analyzed the decision-making process and provided an insider’s view of ‘who gets in’ (Bouchard 2000; Foster 1998; Hawkins 1988; Pratt 2005; Satzewich 2015); the present article thus re-examines the issue in the context following the introduction of automation and AI in the decision-making process.

In line with other issues managed backstage by governments, the process of introduction and use of automation and AI technologies in Canadian immigration has remained largely undisclosed (Molnar and Gill 2018). To overcome this opacity and secrecy, we performed an in-depth content analysis of a large body of governmental documents. Most were obtained through Access to Information (ATI) requests, a powerful source of data collection to enhance transparency in government (Walby and Luscombe 2020). Both public and unpublished documents provide unique insights into automation and AI tools for visa officer decision-making.

This article argues that the administrative borders of Canada are being transformed through technology, and new human–technology interactions, for the processing and assessment of immigration applications. More specifically, it reveals how the introduction of ADSS tools modifies the process of determining 'who gets in' by displacing and altering by whom, where, when and how discretion is exercised. To support this argument, the article is divided into four parts. The first part presents our theoretical and conceptual framework. The second part describes our investigative method. The third part provides a brief review of recent tools introduced in Canada for processing visa applications. The fourth part reviews the use of Chinook as a case study for the repercussions on the decision-making process for temporary residence applications, clarifying how human discretion is affected. In conclusion, we discuss the implications of introducing assisted decision-making in determining 'who will get in' to Canada, acknowledging the need to consider how the application assessment process cannot be separated from substantive decisions. Theoretically, we argue that these changes reconfigure the administrative borders, characterized by enhanced opacity and complexity. This significant transformation of administrative processes for assessing immigration candidates has profound consequences for bureaucrats who process applications and for candidates to immigration alike.

## Theorizations of ADSS and the transformation of administrative borders

Recent scholarly work on the transformative potential of ADSS in public administrations addresses issues of efficiency and effectiveness, as these tools harness the power to reduce waiting times and cut costs down, but may also lead to massive errors (Baynes 2019; Deck 2023; Molnar and Gill 2018; Oluwasanmi 2021). Concerns with bias and fairness are much discussed pitfalls as the use of algorithms can lead to discriminatory outcomes. In addition, transparency and accountability stand as major issues that require monitoring, particularly in the context of public–private partnerships (Chartier-Edwards et al. 2024).

### Administrative borders and SSDC migration management

This article harnesses and revisits the concept of externalized borders put forward by critical border studies, which unpacks the increased reliance on ‘remote border control’ strategies by states in matters of forced migration (FitzGerald 2020). These studies show that receiving states have recourse to legal, technological, and administrative tools that externalize the control of migration outside their borders. We mobilize this concept to examine how automated, remote control strategies are integrated in the assessment of candidates to border entry for the purpose of temporary immigration.

A first attempt at remote control bordering theorization was successfully made by Haince (2011) who wrote of upstream borders. She showed how the spatiality and temporality of immigration selection constitute bordering practices that aim to select the perfect immigrant. This logic is deeply altered in a digitalised, automated immigration system. With bordering practices involving automation and AI are enacted at the file processing and selection stage, administrative borders are pushed to a new level, both in their thickness, remoteness and opacity. In relation to these changes, more recent theoretical developments (Sasser 2022; Kaun 2025) put temporality center stage in the analysis of the effects of automation. Amoore’s notion of the ‘deep border’ invites scholars to consider how algorithms ‘are reordering what the border means’ (Amoore 2024: 2) and how borders must be ‘spatially reimagined’ (p.1).

In conjunction with theoretical proposals on technologies and borders, scholars have framed the study of the introduction of technology within the migration management literature. Reviewing how AI will transform migration management, Beduschi (2021) concludes that the ‘AI divide could contribute to deepening the asymmetries between states insofar as international migration management is concerned’, and argues that it could reinforce previous practices, including unlawful or problematic ones. In legal studies, Molnar and Gill’s pioneer work (2018) identified refugee rights and privacy concerns of the introduction of AI for migration management in Canada. Their analysis regarding fundamental rights and border control mechanisms concluded that humanitarian immigration is a high-risk laboratory for the implementation of automated decision-making in Canada. Canada’s adoption of AI for its migration management has been cited as a study of the risk mitigating strategies of public administrations (Kuziemski and Misuraca 2020). More recently, Molnar’s analysis (2024) of AI technologies in border control at the global scale argues that a lucrative industry of migration control serves to exacerbate discrimination and power asymmetries between migrants and people making decision about how to manage them.

Nalbandian’s groundbreaking work (2021) reveals many of the operational details of Advanced Analytics software, IRCC’s best-known AI solution. Analyzing interviews with visa officers and public government documents, she shows it is unclear how the Canadian public administration draws lines of accountability after automated decision-making was introduced in Canada’s Temporary Resident Visa (TRV) stream (Nalbandian 2022). Brunner and Tao (2024) examined issues related to the introduction of automation and AI for the processing of study permits in Canada and underscore how these new technologies impact the governance of international students in complex ways to that extent that ‘technological advances in migration may govern not just international students but also, to some extent, education systems themselves’ (Brunner and Tao 2024, p. 282).

This article also builds on the literature on the introduction of automated decision-making systems in public administrations, its impacts on decision-making processes and the use of discretion among bureaucrats. We refer to discretion as “the freedom, power, authority, decision or leeway of an official, organization or individual to decide, discern or determine to make a judgment, choice or decision, about alternative courses” (Gelsthorpe and Padfield 2003, p. 3). Following Lipsky’s seminal analysis of street-level bureaucracy, the literature on discretion discusses the introduction of screen-level bureaucracy (Bovens and Zouridis 2002), whereby computers are omnipresent in bureaucrats’ day-to-day work and face-to-face interactions with clients are becoming less frequent. The following stage, system-level bureaucracy, is when automated systems treat files in batch rather than bureaucrats assessing cases one by one. In this context, the screen-level bureaucrat handling individual cases, such as immigration officers, could be marginalized to be replaced by specialists in computerized systems, policy experts, and managers setting goals for these systems, and customer support focused on handling complaints and helping the public interact with computer interfaces, often called portals.

As automation software organizes which information is displayed and how it is displayed for officers, and makes some decisions, sub-decisions or recommendations upfront, bureaucrats’ work is transformed (Côté-Boucher 2016). As a ‘human-in-the-loop’, a bureaucrat with power to make decisions might only be involved in more complex cases or to confirm automated decisions. Côté-Boucher (2014) showed that the reduction or withdrawal of discretion in the day-to-day work of customs officers at the Canada Border Services Agency transformed their experiences and occupational identity. The limited space imposed by automation on the exercise of human judgment can impact how bureaucrats perceive and perform their work (Paul et al. 2024).

### Discretion in migration management

Borrelli et al. (2023) discuss key insights on recent research on discretion in migration management and provide useful categorizations to better relate our research to other ongoing currents. Notably, they highlight that “state power does not necessarily decrease as a consequence of discretion at the frontlines […] [but rather] transforms into an opaque and informal form of governance that can select migrants according to how desirable they are to host states but without codifying this sorting in law.” (Borrelli et al. 2023, pp.6–7). Opacity is also a known concern of the use of AI and other ADSS for discretionary decision-making (Burrell 2016; Zalnieriute et al. 2021). Furthermore, Borrelli et al. (2025) identified limited research that examines specifically how automation in migration control affects discretion in decision-making and calls for more analyses. This article aims to contribute to this emerging conversation.

The Canadian case is particularly relevant to examine the role of frontline decision-makers in the context of technological changes in the assessment of border entry applications. Canadian visa officers must meet management-enforced criteria of both quality decision-making and performance (Satzewich 2015), which can leave them caught between conflicting sets of norms (Brodkin 2011, Borrelli et al. 2023). One coping strategy when faced with “heavy workloads and managerial pressure” (Borrelli et al. 2023) consists in “moving away from clients” (Tummers et al. 2015), such as limiting direct contact with clients, standardizing procedures, and rationing services (Borrelli et al. 2023). These strategies, in turn, are typical not only of discretionary decision-makers reacting to joint pressures of quality and quantity, but also typical of the transition of street-level bureaucracies to screen-level (Bovens and Zouridis 2002) and system-level ones (Buffat 2015; Busch et Henriksen 2018), in that interacting with clients through increasingly automated computer systems to process applications made through online portals limits interactions with clients and requires standardized intake and processing procedures.

In sum, the reduction of interactions between administrative decision-makers and those affected by their decisions combined with the increased use of computer systems and automation is expected to transform the mainly discretionary work of decision-makers working on temporary resident applications. However, this transformation of the exercise of state sovereignty at the administrative border that is travel authorisation (Torpey 1999) is not expected to eliminate state control, nor to replace discretion with laws and algorithms, but to obfuscate how it operates as well as limit accountability and responsibility. To grasp at these anticipated shifts, it is required to study who makes which decisions, what information they have access to when they make them, as well as where, and by whom, discretion is exercised.

### Establishing a baseline

Satzewich conducted the most detailed fieldwork and study of Canadian immigration officer decision-making (2015) between 2010 and 2012 in 11 of the 44 visa offices within Canadian consular missions. He interviewed visa officers, managers, and other employees, in addition to observing them at work. His work shows how decisions on temporary residence visas (TRVs) are almost exclusively discretionary and complex—and not confined to a simple application of the law and regulations. The Canadian visa approval process required a complex analysis of the individual file but also the consideration of the local geopolitical context, as well as Canadian immigration laws and regulations (Satzewich 2015, p. 23). Moreover, officers must be satisfied (convinced) that the person applying for temporary status will indeed leave Canada at the end of the authorized stay period and will return to their previous country of residence.

His sociological analysis puts forward the notion of constrained discretion. He underscores institutional constraints on officer discretion, which include immigration quotas and targets, pressures to process files quickly and the priority put on fraud detection. Saztewich (2015) identified: departmental orientations, priorities, and policies of the moment (p.40), time constraints (p.140; p.196), the socio-constructed typology of 'normal' immigration cases (p.141) or of socially normal individual behavior (p.55), profiling of problematic cases (pp.148–149), the season of the year and associated migratory fluctuations (p.123; p.192), variations between offices (p.195) and officers (p.56) on the continuum of attitudes of facilitation or interrogation (enforcement) of mobilities, migration thresholds set by the Minister (p.133), as well as the perception of risk (p.50) and each officer's aversion or propensity to take risks of error in their decision-making.

A particular concern affecting visa officers is the fear of making a mistake: either mistaken approvals or mistaken refusals. One mistake they wish to avoid is to accept an applicant for temporary residence who, once in Canada, will apply for asylum (Satzewich 2015, p. 199). Called ‘jumpers’, these candidates can be traced to the officer who accepted them in the first place, thus increasing personal stakes for wrong decisions. Other false positives to avoid are temporary residents who will remain in Canada after their period of authorized stay (overstays), individuals conducting criminal activity or who represent security risks. Fraud and misrepresentation are also on the officers' radar, particularly in certain geographical regions and programs (Satzewich 2015, pp. 147–149). Decision-makers perceive judicial review as the main risk of an erroneous refusal as prospective migrants hold some rights to contest refusals at the Federal Court. To be upheld, a decision must be well justified: "Thus, the notes that officers create and enter into the database are arguably more thorough, involved, and detailed for refusals than for approvals." (Satzewich 2015, p. 136). Officers take particular care to record the notes relating to their decision-making when issuing refusals, which requires time and effort.

Satzewich observed how knowledge and expertise about local practices and realities was key to quality visa office work. Visa officers, with the help of local employees and their local experience and cultural sensitivity, develop an understanding of the types of applications in which fraud is more plausible, making this local expertise crucial to the file assessment process. It can lead to additional verification measures, such as face-to-face immigration interviews, procedural fairness letters that allow a person to respond in writing to the officer's concerns, and more thorough research and investigations, especially when fraud is suspected (Satzewich 2015).

## Data and methods

Our method mobilizes the notion of ‘ethnographic refraction’ in the study of algorithm (Christin 2020). While our focus is not on algorithm, the analysis of the impacts of systems that rely on them may use a similar lens. Ethnographic refraction is defined as the study of interactions between social actors including, in our case, government bodies, software and algorithm designers (private or public), and civil servants interacting with automation software and AI (Christin 2020). The premise of this approach is that systems do not exit in a vacuum, but, rather, are inscribed in dense institutional structures, practices and networks.

The analysis is based on an extensive content analysis of various sources. We classify our sources in two broad types. First, our main body consists of *backstage documents* (Walby and Larsen 2011). It consists of documents obtained following Access to Information requests (ATI in Canada, called Freedom of Information, FOI, in British and American contexts) representing over 17 000 pages of documents related to automation and AI, mostly from IRCC, requested and released between January 2021 and March 2024. We used an iterative multi-stage ATI method whereby careful analysis of one document provided us with the knowledge and language to request other ones. We obtained email exchanges, training materials, user manuals, policy manuals, local visa office instructions and standard operating procedures, reports, public relations lines, data sets, modernization strategies, project development roadmaps, documents related to Algorithmic Impact Assessments, and Privacy Impact Assessments.

In combination to these ATI documents, most of which were partially censored, we also collected publicly available documents, the *frontstage documents* of government (Walby and Larsen 2011). Some of these documents, however, are archived and required significant effort to locate and retrieve. This part of our collection includes federal court filings, public reports, statistical data, and parliamentary proceedings. Key *frontstage documents* included presentations made by public servants working on automation solutions at IRCC (Laferrière and McPherson 2019; Mann and Hersch 2019; Sovani 2019). Because these mention or hint at the existence of *backstage documents*, they informed targeted ATI requests.

Through ATI requests, various versions of instruction manuals for the Chinook software were obtained (IRCC 2021c), as well as documents related to other software, such as Watchtower, Hiraya, and Cumulus. We also identified key actors on these projects; to better understand the work teams they belonged to, we consulted federal public servant registries, such as Government Electronic Directory Services and organizational charts. We requested email correspondences within these teams, and learned about further projects, references to further administrative documents, which we requested through further ATI requests. These smaller-scope ATI requests, following an iterative process, provided a steady flow of new information. After some initial results, slower, wider-scope ATI requests targeting broader documentation relative to specific applications arrived, compensating for some of the information missing in earlier, narrower requests.

We entered our entire corpus of government documents, including 17 149 pages of *backstage documents* (non-public), into a Pinpoint database (MIT Library 2024), which rendered PDFs searchable thanks to Optical Character Recognition without altering originals. We tagged items by document type and thematic content. Close reading of documents revealed themes, making the tagging process emergent and reiterative. We used 53 thematic flags related either to the software a document was about, or to a topic discussed therein.^1^ Consulting the sub-set of documentation pertaining to Chinook (mostly from ATI obtained documents), we used Pinpoint functionalities to highlight and hyperlink relevant passages detailing software features, officer workflows, and efficiency measures that indicated changes to the exercise of discretion. Our study is an example of how social scientists may tap into this invaluable source of data for the study of sensitive topics, including technologies.^2^ Our analysis, however, is limited by its lack of direct information from visa officers who are held responsible for decisions.

## The introduction of automation and AI in Canadian immigrant selection

IRCC argues that increasing volumes of applications and the shortage of resources to process them constituted the context that prompted the progressive introduction, testing and implementation of automation. In 2012, IRCC^3^ managers considered their teams would not be able to process more temporary resident visa applications without affecting the quality of decision-making, unless there was an increase in staff (CIC 2012a; CIC 2012b). Internal data quoted by IRCC indicated that between 2011 and 2019, temporary resident visa application volumes increased by 109%, work permits by 147%, study permits by 222% (Affidavit of Andie Daponte 2021). In return, IRCC says visa office staff increased by only 20% between 2013 and 2017 (Sovani 2019). Other sources indicate that visa office immigration staff levels may even have dropped to as low as 1500 in 2022 (Calvert 2022), as opposed to 1680 in 2011 (CIC 2012). Canada received more than 2.5 million applications for temporary residence in 2016, all categories combined. These figures show high-volume pressure from temporary immigration applications, the lines of business (immigration categories of entry) targeted by IRCC for the introduction of automation tools to manage these volumes, including Artificial Intelligence (AI) (Affidavit of Andie Daponte 2021; Laferrière and McPherson 2019). Application volumes increased significantly, staffing levels stagnated, but what makes a good decision stayed constant, creating competing administrative pressures (quality and quantity) on officers.

### Norms, transparency and accountability

The 2015 addition of Part 4.1 ‘Electronic administration’ to the Immigration and Refugee Protection Act authorized the use of automated systems in decision-making at large, as long as “the system is made available to the officer by the Minister.” (IRPA 2001 paragraph 186.1 (5)). House of Commons discussions of this amendment raised issues of risks of biases, potential need for supervision and of an ombudsman, and the need to protect privacy of biometric data (Canada 2015). Proponents argued that automation projects would reduce costs, increase efficiency and maintain program integrity by identifying fraud more efficiently, all the while reducing waiting times.

With this simple legislative change, IRCC could adopt any ADSS as long as it had the Minister’s approval. The only other significant obligation imposed on IRCC since has been the Treasury Board Secretariat (TBS) Directive on Automated Decision-Making (TBS 2025). Adopted in 2018, taking effect in 2019, and applying to ‘automated decision systems developed or procured after April 1, 2020’, it gives one year for compliance from adoption date by publishing an Algorithmic Impact Assessment (AIA), in the form of responses to a standardized set of questions. From 2015 through 2021, this created a context where transparency was optional, which is in part why our data collection had to rely on Access to Information (ATI) requests.

Following this legislative update, IRCC was empowered to scale up the introduction of automation; many new projects from this point on went without further public or political discussions and were treated as in-house matters.

### Accelerating ADSS developments

The Global Case Management System was implemented in 2012 following digitisation of applications across all lines of business by both IRCC and the Canada Border Services Agency. This system is the “single, integrated and worldwide system used internally to process applications for citizenship and immigration services” (IRCC 2012). It allowed the orderly and centralized collection of data that paved the ground for further automation. In 2014, CIC began to work on automation measures incorporated in the Express Entry platform, as well as more sophisticated predictive analytics systems in test environments, more akin to artificial intelligence (Keung 2017). Initial forays into automation required ministerial instructions and were therefore publicized.

In 2017, a software program called Hiraya with automation features was implemented in the Colombo visa office (Sri Lanka) to accelerate and partly automate the treatment of temporary visa applications, with help from the Manila office where it was previously used (IRCC 2018). Trainings were planned to use the same tool in different offices; later, documents show it was eventually used in the Sydney office, and was used at least until April 2019 (IRCC 2021d). Hiraya leveraged automation features; documents estimate that officers could process more than 50 requests per day (less than 10 min per request), sometimes without even having to consult the attachments (IRCC 2018). Officers worked faster when files were grouped and refused in bulk, or pre-assessed by local employees (IRCC 2018).

In 2018, IRCC launched the Advanced Analytics pilot project to triage temporary residence visa applications received digitally from China, and later India. Because it was released before the aforementioned TBS Directive on Automated Decision-Making came into effect, no AIA was provided. IRCC itself revealed the existence of the tool to the public in 2019 (Laferrière and McPherson 2019). This AI tool triages applications into three groups – Tier 1 comes with automated eligibility approvals and expedited processing, but the other tiers do not (Nalbandian 2022). An officer makes the final decision on each request and agrees with the AI in at least 98% of Tier 1 (low risk) cases (Laferrière and McPherson 2019). The pilot project was used continuously until at least January 2020 and may never have been discontinued according to ATI documents we obtained (IRCC 2021g). In 2020, the Advanced Analytics Project was extended to temporary residence visa applications received by mail from Visa Application Centers (IRCC 2020b). The AIA for the ‘Advanced Analytics Triage of Overseas Temporary Resident Visa Applications’, or Rest of World (ROW) Model, which presents as the successor to the Pilot, was published on January 21 2022, nearly four years after the initial Pilot was rolled out (IRCC 2022a).

Other automation and AI tools include Spouse or Common-Law Partner in Canada Advanced Analytics, Watchtower, Lighthouse, Starling, Cumulus, and a slew of ‘Officer Rules’ office-specific triage techniques. AIAs on most of these tools have not been conducted or if so, not made available to the public, since they were implemented prior to the 2019 Directive’s application. Those published contain very little information relevant to evaluate risks of bias, privacy leaks, or significant changes in administrative procedures and decision-making. Furthermore, these AIAs are self-evaluations, and not third-party audits.

This brief summary of the array of ADSS introduced by IRCC since 2015 indicates that there is a profound transformation underway with the introduction of greater automation in immigration application processing throughout IRCC offices. While public discussion of the use of ADSS by IRCC has revolved mostly around the use of Chinook (Chartier-Edwards et al. 2024) and Advanced Analytics (Nalbandian 2022), the analysis of our corpus of ATI has shown a much broader scope of application of these ADSS solutions, present across all lines of business at IRCC, and not all of them revealed to the public, nor to parliament.

## How the introduction of ADSS transforms the exercise of discretion: the case of Chinook

In this section, we will focus on a particular tool, Chinook, to illustrate how it transforms the exercise of discretion by visa officers. The tool was rapidly deployed and authorized for use in all visa offices since 2018, and so was the primary target of our data collection as of 2021. We use it as a case study also because it is the tool for which we obtained the most information to date. Moreover, because it processes temporary immigration applications of candidates overseas, it raises particular issues with respect to access to justice in case of refusal.

Chinook started as a desktop software, adding Visual Basic programming to Excel for a layer of automation and enhanced interface features (Affidavit of Andie Daponte 2021; IRCC 2021b, 2022c). Core functionalities discussed here are common across versions for which documentation was available.^4^ Each office and officer is reputed free to choose to use the software or not, including any version (Affidavit of Andie Daponte 2021). Chinook is modular—some portions of the software may be used independently of others. The tool extracts data from Global Case Management System (GCMS) case files and displays it in a single workbook, allowing rapid actions on multiple applications. Chinook exports any actions and final decisions to the GCMS, which remains the official reference database and registry of record for immigration files, deleting any Chinook transit data in the process (IRCC 2020a).

Chinook is divided into five main modules, some of which can be updated independently from the overall environment and can have their own version numbers (IRCC 2019d). Module 1 is for managers, it is used for the creation of application groups and the assignment of workloads to employees; Module 2 is used for pre-evaluation by local employees or visa officers as well as for planning actions prior to the decision (such as requests for biometric data, medical examinations or scheduling an interview); Module 3 is used for decision-making, including massive decision-making for all members of a group (bulk processing); Module 4 is used for post-decision process management; Module 5 is used for risk indicator management and ‘local word flags’ (Affidavit of Andie Daponte 2021; IRCC 2022c).^5^

As IRCC specifies in its internal document Chinook 101,^6^ the purpose of the Chinook ADSS from its conception was to increase officer productivity (2022c). It was designed in January 2018 to replace various file processing software tools, developed locally and separately, such as Hiraya, used in Manila, Colombo and Sydney, Noma used in London, and Smartbook used in Abu Dhabi, London and Ankara, as well as a slew of similar tools developed in other offices (IRCC 2019a; 2021d; 2022d, p. 3).

Importantly for the present analysis, IRCC maintains that “Chinook does not fundamentally change the way applications are processed” (IRCC 2022c, p. 1). IRCC advances that Ministry officials always review all the information submitted by clients to ensure informed and fair decision-making, the use of Chinook does not result in “a less thorough review of applications, nor does it change the way decisions are made, it simply offers a consolidated view in a more user-friendly way, instead of a multiple screen layout of the application’s information” (IRCC 2022j). In the following subsections, we put these assertions to the test of close scrutiny in light of the evidence we gathered and analyzed. We refer to ADSS systems in general and illustrate some of the features specific to the case of Chinook.

### Systems alter the analysis process of files

The introduction of system-level bureaucracies transforms the way files are analyzed. Systems triage, fragment, and reorganize the analysis process. Chinook’s features alter the way temporary immigration applications are analyzed in three ways: alterations to processing affected by risk flagging or triage levels, group or bulk processing, and limited or no consultation by officers of attached documents submitted with applications.

#### Triaging and risk flagging alters processing

First, many tools interact with applications processed in Chinook, one being Advanced Analytics (AA), an AI. AA triages files according to risk levels in tiers that require attention from visa officers. Tier 1 files receive automatic eligibility (IRPR, 179 b) approvals. From January 2018 to March 2020, 65.8% of refusal reasons provided for a TRV refusal were eligibility refusal (IRCC 2022i). Because of this, an automatic approval of eligibility ensures a very high probability of subsequent overall approval (IRCC 2022i). In contrast, files in Tier 2 or 3 require greater examination from an officer. Data from the AA Triage Pilot in China and India showed that eligibility decisions did vary not only by Tier, but by office as well (IRCC 2020b).

The AA triage tools may class a file in Tier 2 or Tier 3 because the algorithm identified a risk factor or a prediction of “post-approval adverse events” (IRCC 2022g). This can include the predicted risks of overstays, asylum claims, security threats, and crime (IRCC 2022g, p. 4), meaning that IRCC’s Advanced Analytics Triage software, among other things, contains components akin to predictive policing algorithms, a sector of AI with well-documented bias concerns (Joseph 2024; Richardson et al. 2019; Flores et al. 2016).

In the case of Tier 3 files, considered higher risk or complexity cases, or those marked with other, unrelated Chinook Module 5 Risk Flags, it is evident that the work done by the algorithm to label the case in such a manner prior to the intervention of the officer would make the officer aware and wary of risks. Officers could read suspicion into the file (Pratt 2010; Oorschot 2021) as part of this triaging. This puts a considerable burden on the applicant to demonstrate the legitimacy of his or her application given the high-risk flag already associated to their application—and retroactively so. Because officers must be satisfied before approving applications, this suspicion could lead to refusals or to longer verifications. Applicants are not informed of triage groups or risk flags applied and are not necessarily given an occasion to provide clarifying or complementary information to prevent the refusal or the processing of their file be grinded to a halt, leading to long waiting periods for elaborate security checks, a currently growing problem in Canadian immigration (CBSA 2023).

Officers reviewing files could be affected by risk levels in their assessment. Humans with time constraints and limited information tend to believe computer assessments—this is called automation bias (Boken and Rieke 2018). For these Advanced Analytics triage, labels have the objective to direct officers’ attention and time to certain files over others because of their inherent content—be it risk or complexity or a blend of the two. The expected outcome is to give officers more time to assess certain files carefully. For systems that guide decision-makers, the fact that they make quality recommendations can actually reinforce the automation bias and strengthen its effect over time (Jackson et al. 2019).

Therefore, the expected outcomes of triaging in this way mean that Tier 1 applications can be processed faster and with higher expected levels of approval thanks to automated positive eligibility decisions; *a contrario*, Tier 2 and 3 applications are expected to take longer to process and have lower levels of approval.

Overall, the tagging of applications with triage tiers, which are known stand-ins for complexity and risk levels, as well as additional processing instructions riding on Risk Indicators, could reduce the officer’s level of responsibility toward the assessment of the evidence on file, process discretion and the final decision on a file. Opacity of computer systems—such as not knowing why a specific file was placed in a Tier or why a Risk Flag or Local Word Flag was attached to it—hamper the possibility of officers to exercise unbiased assessments or to override system recommendations, and as design choices take precedence over officer judgment, the space for officer discretion is reduced. Some of that discretion is transferred to programmers, managers, and those responsible for Module 5. Decisions considered less conventional or bold, responding to unanticipated situations and conjunctures, and seeking to facilitate access for migrants perceived as desirable (Satzewich 2015) may no longer have space to emerge on the front lines.

#### Bulk processing shifts the salient information in focus

Second, working with Chinook for bulk processing shifts the focus from file particulars to group commonalities. IRCC affirms in several places that Chinook increases productivity with efficiency gains, sometimes quantified from 18 to 30% (IRCC 2022c, p. 9). Chinook therefore pursues an optimization objective already initiated with previous software that it must supplant, in line with its modernization plan (CIC 2012).

Chinook, and Hiraya before it, was designed to be able to optimize decision-making times via bulk processing. For bulk processing to be executed, it is necessary for applications to be grouped based on similarity; officers must have identified in these applications common factors that render them confident and satisfied of a common decision for all of them.^7^ “Module 3 is organized in such a way that efficiencies of scale can be produced by processing like cases together and facilitating grouping in the finalization process.” (IRCC 2021a, p. 70). The same would be true of bulk refusals: those processed collectively would require them to have common refusal grounds.

By building upon Excel as a backbone and leveraging its built-in sorting and filtering capacities (IRCC 2022b), Chinook allows officers to, for example, select all study permit applications destined for Québec, and refuse all those without the provincially issued *Certificat d’Acceptation du Québec* (IRPR 2002, paragraph 216 (3))*.* This approach would save the officer time, but includes error risks as it may lead to faulty refusals. For example, candidates with a scholarship from the Canadian Francophonie Scholarship Program (Global Affairs Canada 2017) are exempted from providing that document (IRCC 2023a), but this workflow could refuse them. As these cases would be rare, officers may gain efficiency nevertheless despite increased error risks. Satzewich had already noted that managers appreciated and knew that efficient visa officers tolerated riskier decision-making (2015). By working on similar applications in groups, officers would be less aware of details and of higher error risks on individual files, and even the more risk-averse may be emboldened to efficiency gains.

This process prioritizes what applications have in common by grouping applications and allowing bulk processing, rather than providing officers with the means to focus on the particulars of each case. This shift is specifically why the US Network of Visa Offices has refused to adopt Chinook Module 3 in its workflow (IRCC 2019a). In a draft mission report (IRCC 2019b), IRCC observed that “caseloads processed by U.S.-based IRCC offices are too specialized and/or complex; not homogenous;” (p.2), limiting the possibility of focusing on similarities. In conclusions to the report, they clarify that “[because] of the diversity seen in applications, leaders could hardly take advantage of the grouping function to action refusals on the same grounds.” (p.3–4). Overall, US network applications required to be handled with care, had overall lower volumes, and non-homogeneous “demographics” (p.4). Not being able to focus on similarities between cases for bulk processing efficiency gains, they chose not to adopt Chinook to continue working on the particulars of each case—especially because of their “very specialized—and often high profile—clientele” (p.4), referring to diplomats and US officials. This illustrates the rationale displayed in one of the rare cases where Chinook was not adopted in visa offices, and, *a contrario*, why it may be so attractive to adopt it elsewhere. It further shows that Chinook Module 3 offers limited efficiency gains outside of bulk decision-making.

Despite the fact that the IRPA (2001) now allows IRCC officers to use an approved ADSS to apply the law, nothing in the law nor the regulations specifically allow them to base the decision in one file on the similarities it has with other files. For applicants wishing to challenge such decisions, the destruction of grouping information and other forms of transit notes from the Chinook system may complicate their ability to show the flaw in the decision-making process, and the “systematic daily deletion of all material generated by processing technology may not reflect best practice” (Mehrara v. Canada (Citizenship and Immigration) 2024 FC 1554).

#### System limitations alter information available for decisions

Third, these systems operate on limited information when they are fully automated, and even when they are only supports to officer decision-making, they only display partial information to officers. When Advanced Analytics triages an application into a tier, it does so before pre-assessment (IRCC 2022h). Pre-assessment, a process usually carried out by local employees, extracts information from attachments and summarizes it in pre-assessment note text fields (Affidavit of Andie Daponte 2021) which are then structured and digest for an ADSS. Without considering the content of document submissions, it bases that determination only on GCMS data and application form answers that have been digitized thanks to two-dimensional barcodes (CIC 2012a; CIC 2012b; IRCC 2012). In the case of Chinook, which can display structured data from the GCMS database application history and application form content of the files being reviewed, it does not provide the officer with the possibility of considering documents attached to files (Affidavit of Andie Daponte 2021), which IRCC calls edocs (IRCC 2021f), as these are not automatically rendered into structured data. Officers should read edocs in full (IRCC 2015), but may not be allocating enough processing time to do so (IRCC 2020a; 2022f, 2021f), and may either rely on pre-assessment notes, or only read enough edocs to render a decision (IRCC 2019c).

When candidates to temporary immigration submit an application, they may attach hundreds of pages of such supporting evidence in the form of documents: bank statements, study plans, invitation letters, job offers, bank statements, marriage certificates, birth certificates, support letters, property titles, diplomas and transcripts, etc. (Bashirahishize 2022; Guay 2023). It remains an obligation for the decision-maker to be aware of all of the evidence on file: “It is important to highlight that, ultimately, it is the migration officer's responsibility to assess all the documentation necessary to make a decision’’ (IRCC 2019c, p. 3). In the pre-automation era described by Satzewich and others, visa officers or locally engaged staff consulted these documents in pre-assessment. With automation, whether all attached documents are given due consideration, and by whom, remains unclear. This raises issues of procedural fairness, which requires applicant submissions be heard (read), in full, by the same person who will render a decision (IRCC 2015).

### Systems alter the temporality of decision-making

The introduction of ADSS have the objective of speeding up processes, reducing backlogs and clients’ waiting times. Chinook’s bulk processing promises to significantly reduced processing times. Triage promises to save time on easy files to allocate it elsewhere.

How fast can visa officers process applications and still render quality decisions? During his 2010–2012 fieldwork, Satzewich analyzed interviews with visa officers and from information he provides, we can infer that they were taking between 3 and 12 min of decision-making per file and still felt pressure to process faster (2015). This likely excludes pre-assessment, and post-decision processing such as note-taking. During this same time period, IRCC managers stated they were achieving peak processing capacity for the number of staff they had, and processing more applications would affect the quality of the decision-making (CIC 2012b). From that point forward, the ratio of temporary residence applications to be processed to the number of officers increased further (Affidavit of Andie Daponte 2021; Sovani 2019).

Internal IRCC data shows that daily targets for overseas study permit processing would be an average of 7.5 min per application, but this is an upper limit: “the time and motion demonstrated that the vast majority of files on average take less time than the new expectations outlined above. Based on this, management feels that these expectations are fair.” (IRCC 2023a, b in Meurrens 2024). Project Peacock, a test of early, less automated Chinook processing speeds, measured officers rendering 105 decisions per day—meaning an average of 3.7 min per application (IRCC 2020a). A subsequent time and motion analysis showed some Chinook users had average processing times under 102 s per file (IRCC 2022f).

Finally, IRCC’s approach to slower processing times, rather than improving them uniformly, has been to use automation and AI triage to prioritize the processing of certain applications over others. AI triage and its potential for automated eligibility findings, by processing “easier” applications faster, have led to an increased disparity between faster and slower applications. IRCC does not proactively publish this differential in processing times. In 2010, the differential between the fastest and slowest application up to the 80th percentile of processing times was at most 20 days (CIC 2012a). Today, it can be over one year (IRCC 2023b). This disparity in processing times is based on hidden factors, not revealed to the public, parliament, applicants, nor even to visa officers. These results echo Susser’s work on the speeding-up of decision-making processes. By analyzing temporality, he questions how decision-makers (here, visa officers) live up to values of transparency, accuracy and fairness (Susser 2022: 2)?

### Systems alter the spatiality of the decision-making process

Satzewich’s study underscored how "local knowledge”, localized understandings of culture, society, institutions and practices, were center stage to decision-making. Locally engaged staff were seen as local knowledge experts. Adopting Chinook requires standardizing practices and procedures across different visa offices (IRCC 2022d, p. 3, 2022e, p. 5). It also aims to standardize approaches to triage and prioritization of files, and to facilitate workload sharing between different visa offices (IRCC 2022d, pp. 22–23). These objectives are thus counter to the assumption that local expertise, or localized processing, is key to the proper (pre-)assessment of a file.

By facilitating the sharing of workloads between different visa offices, the possibility that a file will be processed by an officer located elsewhere in the world, without the same access to local staff and expertise, increases. This physical relocation of the decision is not necessarily communicated to the applicant and his or her representative. If noted in GCMS, this information sometimes disappears (IRCC 2020d) because instructions to visa officers at a secondary office or Risk Assessment Unit tell them to erase their assignment to the file in GCMS after performing their tasks (IRCC 2021f, p. 249). Legal practitioners have noted that refusal letters increasingly omit to indicate the office rendering decisions (Desloges and Sawicki 2021).

Further, the transfer of decision data from Chinook to GCMS does not contain all Chinook data, but only the final decision and the preformatted refusal notes provided by Chinook to justify this decision (AQAADI 2021; Tao 2021; Mehrara v. Canada 2024). Without a proper audit trail, it is impossible to establish with certainty whether this relocation has taken place, and whether the decision was made by an officer with local expertise, or not.^8^

## Conclusion

The research question this article sought to address was how do automation and AI systems affect the way decisions are made in Canadian immigration as to ‘who gets in’? The analysis makes three scholarly contributions.

First, theoretically, this article contributes to border studies by advancing the concept of administrative borders and showing how they are being reshaped through the integration of automation and AI in immigration decision-making. Drawing on critical border theory, we contribute to existing literature that borders are increasingly enacted through bureaucratic and technological infrastructures. By analyzing a tool like Chinook, we demonstrate how discretion—once exclusively exercised by frontline officers—is redistributed across software systems, institutional actors, and opaque algorithmic processes. This reconfiguration results in thicker, more remote, and less transparent borders, where decisions about “who gets in” are shaped by automated triage, algorithmic risk assessments, and bulk processing. We thus re-conceptualize the border as a dynamic administrative space where sovereignty is exercised through digital systems, raising new concerns about accountability, fairness, and access to justice. Moreover, our results about the reconfiguration of the temporality and the spatiality of decision-making contribute to theoretical propositions about how automated decision-making systems in migration management calls for new ways of conceptualizing the border (Amoore 2024).

Second, this article makes a key sociolegal contribution by challenging the conventional separation between substance and process in administrative decision-making. While Canadian administrative law traditionally focuses on the outcome of decisions rather than the processes behind them (Craig 2006; Hagshenas v. Canada 2023), we show that the design and operation of these digital systems shape the exercise of discretion in ways that are legally and ethically significant. By uncovering the procedural transformations introduced by automation, we call for a rethinking of legal accountability and procedural fairness in the age of algorithmic governance. More research is needed to fully assess the legal implications of the changes under way.

Third, this article offers a significant methodological contribution through its use of ethnographic refraction, a framework that enables the study of algorithmic systems by examining their effects within institutional and social contexts. Rather than attempting to access or audit the algorithms directly, we analyze how these systems shape bureaucratic discretion and workflows in Canadian immigration. This is achieved through an extensive and iterative Access to Information (ATI) strategy, which yielded over 17,000 pages of internal government documents. By combining an ethnographic approach to administrative processes with documentary analysis, the article demonstrates how researchers can investigate opaque, technology-driven governance practices and offers a replicable model for studying algorithmic systems in other public sectors.

## Data Availability

All the unpublished documents obtained following a request for access to information made to the Government of Canada can be made available to other people upon request to the Government of Canada. Our reference list contains the details about the documents we obtained. This information would allow another person to request access to these documents.

## Footnotes

1 This is only one study of a larger research project conducted with several collaborators.

2 For a methodological reflection on the use of ATI documents for the study of Canadian drone stakeholders, see Bracken-Roche (2019).

3 In 2012, IRCC was called CIC. Other than a name change in the department, there is continuity of the scope and mandate of the department over this period.

4 Chinook + , Chinook 1.5, Chinook 2.0 and Chinook AWS from documents, as well as the Chinook Tab integration within GCMS. Recent court cases refer to GCMS notes detailing the use of Chinook Version 3 + (Mehrara v. Canada (Citizenship and Immigration) 2024 FC 1554).

5 Modules 6 and 7 present in earlier versions, focused on Quality Assurance (Reporting) and a Toolbox, appear to have been dropped from later versions, with plans to reintegrate Module 6 on the cloud-based (browser-based) environment once Chinook reaches version 2.0 (IRCC 2020c).

6 We thank our collaborators, lawyers Andrew Kolton and Wei Will Tao, for sharing this document and others to help build our collective ATI database.

7 “Once all groups are closed, you can use the All Files Query String to create a super group for all. your approval files." IRCC 2022a, b, c, d, e, f, g, p.14, Chinook Decision Maker Manual, 1A-2022–79418.

8 However, this erasure of the local skills of officers is possibly tempered by the existence of Module 5 of Chinook, which allows managers to create 'local word flags', indicators that provide, in Chinook, specific instructions for cases that are automatically spotted by the presence of a word or a sequence of words (IRCC 2021e). Expertise in the form of discretion is replaced with standardized, automated processes – devoid of discretion.
